# Urbanization Facilitates Bullfrog Invasion Success and Exacerbates Native Amphibian Declines: A Natural Experiment From the COVID‐19 Lockdown

**DOI:** 10.1002/ece3.72633

**Published:** 2025-12-16

**Authors:** Jiayi Shi, Keming Wang, Zhirong He, Qingyan Sun, Meiting Liu, Suyue Wang, Chunna Zhang, Yujia Sun, Na Zhao, Supen Wang

**Affiliations:** ^1^ Collaborative Innovation Center of Recovery and Reconstruction of Degraded Ecosystem in Wanjiang Basin Co‐Founded by Anhui Province and Ministry of Education, School of Ecology and Environment Anhui Normal University Wuhu China; ^2^ Beijing Key Laboratory of Emerging Infectious Diseases, Institute of Infectious Diseases, Beijing Ditan Hospital Capital Medical University Beijing China; ^3^ Anhui Provincial Key Laboratory of Biodiversity Conservation and Ecological Security in the Yangtze River Basin, College of Life Sciences Anhui Normal University Wuhu China

**Keywords:** amphibian decline, biological invasion, biotic homogenization, COVID‐19 lockdown, human disturbance gradient, *Lithobates catesbeianus*, species tolerance, urban ecology

## Abstract

Urbanization and biological invasions synergistically threaten global biodiversity. The COVID‐19 lockdowns created a unique quasi‐experimental reduction in human mobility. We leveraged this period to conduct paired field surveys during both suppressed and resurgent human activity across urbanization (urban/rural) and invasion (invaded/uninvaded) gradients in China's Yangtze Basin, assessing the invasion dynamics of the American bullfrog ( *Lithobates catesbeianus* ). Key findings reveal three core patterns: (1) Urbanization facilitates invasion—bullfrog densities averaged 1.69 times higher in urban than rural habitats, a pattern unexplained by measured habitat variables; (2) Invasion impacts are species‐specific: *Bufo gargarizans* abundance increased in invaded sites (with the stronger response in urban areas), while four native frog species ( *Pelophylax nigromaculatus* , *P. plancyi* , *Fejervarya multistriata* , *Microhyla fissipes* ) declined, with *P. nigromaculatus* , *P. plancyi* and *Microhyla fissipes* experiencing intensified suppression in urban landscapes; (3) The reopening and resumption of human activity drove a 12.1% greater increase in bullfrog densities in rural versus urban areas, suggesting that rural establishment is dispersal‐dependent. Mechanistically, urbanization accelerates invasions through anthropogenic dispersal networks and permanent water sources, while habitat fragmentation forces native species to aggregate, intensifying competition and disease transmission. These findings underscore the need for differentiated urban–rural conservation strategies to mitigate invasion impacts.

## Graphical abstract

This study investigates how urbanization amplifies the invasion of American bullfrogs and exacerbates declines in native amphibians in China's Yangtze Basin, using the COVID‐19 lockdown as a natural experiment. It finds that urban areas have higher bullfrog densities, likely due to human dispersal networks and permanent water sources, while native species face intensified competition and disease transmission. The research underscores the need for differentiated urban–rural conservation strategies to mitigate invasion impacts.

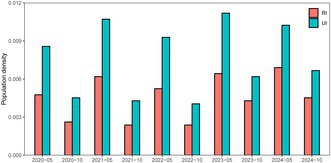

## 1 Introduction

The modern era presents a host of human‐related challenges to the world's ecosystems, with two of the most pervasive threats stemming from the growing urban footprint and the increasing spread of invasive species (Corlett et al. 2020; Pelletier and Coltman 2018; Pyšek et al. 2020). Urbanization continues to increase worldwide, with projections indicating that over 5 billion people will reside in urban areas by 2030, potentially tripling the spatial extent of urban landscapes (Seto et al. 2012). This rapid expansion has precipitated profound environmental transformations (Beninde et al. 2015). Urbanization exerts multifaceted impacts on climatic conditions (e.g., urban heat island effects) (Krayenhoff et al. 2018), atmospheric chemistry (e.g., increased CO_2_ and nitrogen deposition) (Lewis 2018), and biodiversity (Jung et al. 2019), and has been identified as one of the primary drivers of global biodiversity degradation (Alberti 2015; Beninde et al. 2015; McKinney 2008; Sullivan et al. 2017). Given amphibians' heightened sensitivity to environmental and ecological changes (Gersava et al. 2020; Vershinin et al. 2015), they experience more pronounced negative effects from urbanization‐induced environmental alterations compared to other vertebrate taxa (Mazgajska and MazgajskiI 2010). The principal drivers of amphibian population declines in urbanizing landscapes include land‐use changes that reduce habitat area, cause habitat fragmentation, induce habitat segregation through surrounding landscape modification, and degrade habitat quality (Becker et al. 2007; Gibbs et al. 2005), while potentially facilitating the introduction of non‐native species (Goddard et al. 2010; Tsuji et al. 2011).

The frequency, magnitude and impacts of biological invasions are escalating globally (Pyšek et al. 2020; Seebens et al. 2017), and invasive species are now a major component of global change that can alter ecosystem functioning (Lopez et al. 2022; Murphy and Romanuk 2013). Due to the nature of how invasive species are relocated outside their native ranges (e.g., transportation networks, pet trade, or as a biocontrol for pests), many biological invasions are innately linked to human‐dominated landscapes (Pyšek et al. 2020). Human activities can help invading species overcome these barriers by supplying novel niches (Tingley et al. 2013), eliminating native predators and competitors (Liu et al. 2014), or facilitating long‐distance dispersal (Wilson et al. 2009).

Invasive species tend to favor disturbed environments, including urban areas (Cadotte et al. 2017; McKinney 2008). The disturbance hypothesis posits that non‐native species exhibit higher invasion success in highly disturbed ecosystems compared to relatively undisturbed ones (Jeschke and Heger 2018). Human disturbance drives the decline of many species, both directly and indirectly. Nonetheless, some species do particularly well around humans. One mechanism that may explain coexistence is the degree to which a species tolerates human disturbance (Samia et al. 2015). As environmental changes driven by human activities like urbanization are often rapid and intense, it is expected that the tolerance thresholds of many species will be exceeded (Hendry et al. 2007; Sih et al. 2011). Indeed, a common outcome of urbanization is a sharp decline in species diversity (Shochat et al. 2010). Within this filtering process, native species may fail to adapt to the altered environmental conditions, thereby creating resource opportunities for invaders (Shea and Chesson 2002). Conversely, some invaders may possess key adaptations to human‐disturbed environments that native species lack, granting them the advantage needed for successful invasion (Shea and Chesson 2002). This aligns with the view that the successful establishment of invasive species is often associated with broad environmental tolerance (Marvier et al. 2004; Pettitt‐Wade et al. 2015). Consequently, the differential responses of fauna to urbanization reflect disparities in their adaptive arsenal for coping with environmental change (Sol et al. 2013), ultimately leading to the decline of sensitive native specialists and the expansion of tolerant invaders and generalists. The American bullfrog ( *Lithobates catesbeianus* ) is a highly detrimental invasive species, listed among the100 World's Worst Invasive Alien Species by the IUCN Invasive Species Specialist Group (ISSG) (Lowe et al. 2000) and recognized as the only vertebrate in China's first official list of invasive alien species. It causes severe ecological damage through trophic suppression, resource competition, and disease transmission (Kats and Ferrer 2003; Kiesecker and Blaustein 1998; Kiesecker et al. 2001; Lawler et al. 2001).

The SARS‐CoV‐2 pandemic triggered an unprecedented global perturbation in human mobility, creating a unique, large‐scale natural experiment (Rutz et al. 2020). This sudden, worldwide shift in human activity provided a unique opportunity to investigate the effects of large‐scale anthropogenic changes on wildlife (Manenti et al. 2020). We leverage this opportunity to test the hypothesis that species tolerance filters invasion outcomes along human activity gradients. To curb viral transmission, China implemented the world's first COVID‐19 lockdown in Wuhan on January 23, 2020, followed by nationwide strict containment policies, including case isolation, travel restrictions, closure of entertainment venues, and bans on public gatherings. These large‐scale interventions profoundly and abruptly altered human activity patterns and daily behaviors globally (Huang et al. 2020). Subsequently, China lifted mandatory quarantine measures on January 8, 2023, leading to a gradual recovery of typical human activity levels. China's stringent lockdown and subsequent reopening created a quasi‐experimental setting. It specifically enabled us to quantify the bullfrog's invasion dynamics under contrasting intensities of human activity (Lockdown vs. Reopening) and to assess whether its population trajectories differed between urban and rural landscapes during the recovery phase. This situation provided a rare opportunity to isolate the effect of changes in human activity levels, helping to validate its role as a mechanistic filter in biological invasions.

The lower Yangtze Basin—a global urbanization hotspot where bullfrog populations are well‐established—serves as an ideal model system for our research. We integrated this unique anthropogenic perturbation with a paired study design spanning gradients of urbanization and invasion status. Our framework addresses three central questions. First, we investigate the spatiotemporal pattern of bullfrog invasion across the urban–rural gradient and assess the extent to which this pattern is determined by local habitat variables versus broader mechanisms linked to human disturbance. Second, we ask how bullfrog invasion impacts the native anuran community and whether these impacts are uniform or vary significantly between species and between urban and rural contexts. Third, we examine how the post‐reopening resurgence of human activity influenced bullfrog densities and whether this effect differed between urban and rural populations, which may reflect underlying differences in invasion stage or dispersal dependency. Through systematic 5‐year monitoring, this study leverages a unique global event to mechanistically understand how human activity gradients filter species based on their tolerance, ultimately determining the success and impact of biological invasions.

## 2 Materials and Methods

### 2.1 Study Area and Experimental Design

This study was conducted in the border region of Wuhu and Ma'anshan, located in the middle‐lower Yangtze Plain. The region features a humid subtropical monsoon climate, characterized by distinct seasonal variations, warm temperatures, abundant rainfall, and long frost‐free periods (Chen et al. 2016). As a core part of the Wanjiang City Belt, it serves as a national‐level industrial transfer demonstration zone and represents the most economically developed and urbanized area in Anhui Province (Han et al. 2011). With extensive water transportation networks and dense railway/highway systems, it functions as a critical corridor connecting central‐western China to the Yangtze River Delta (Lian and Bao 2012). According to the *Anhui Statistical Yearbook (2024)*, Wuhu has a permanent population of 3.756 million, with an urbanization rate of 74.46%. Its built‐up area covers 266.00 km^2^, including 47.47 km^2^ of residential land, 4.00 km^2^ of public facilities, 43.20 km^2^ of transportation infrastructure, and 24.04 km^2^ of green spaces, with a population density of 1809 persons/km^2^. Ma'anshan, on the other hand, has a permanent population of 2.191 million and an urbanization rate of 73.62%, with a built‐up area of 108.59 km^2^ (28.72 km^2^ residential, 1.64 km^2^ public facilities, 11.31 km^2^ transportation, and 4.10 km^2^ green spaces) and a population density of 4688 persons/km^2^.

**FIGURE 1:**
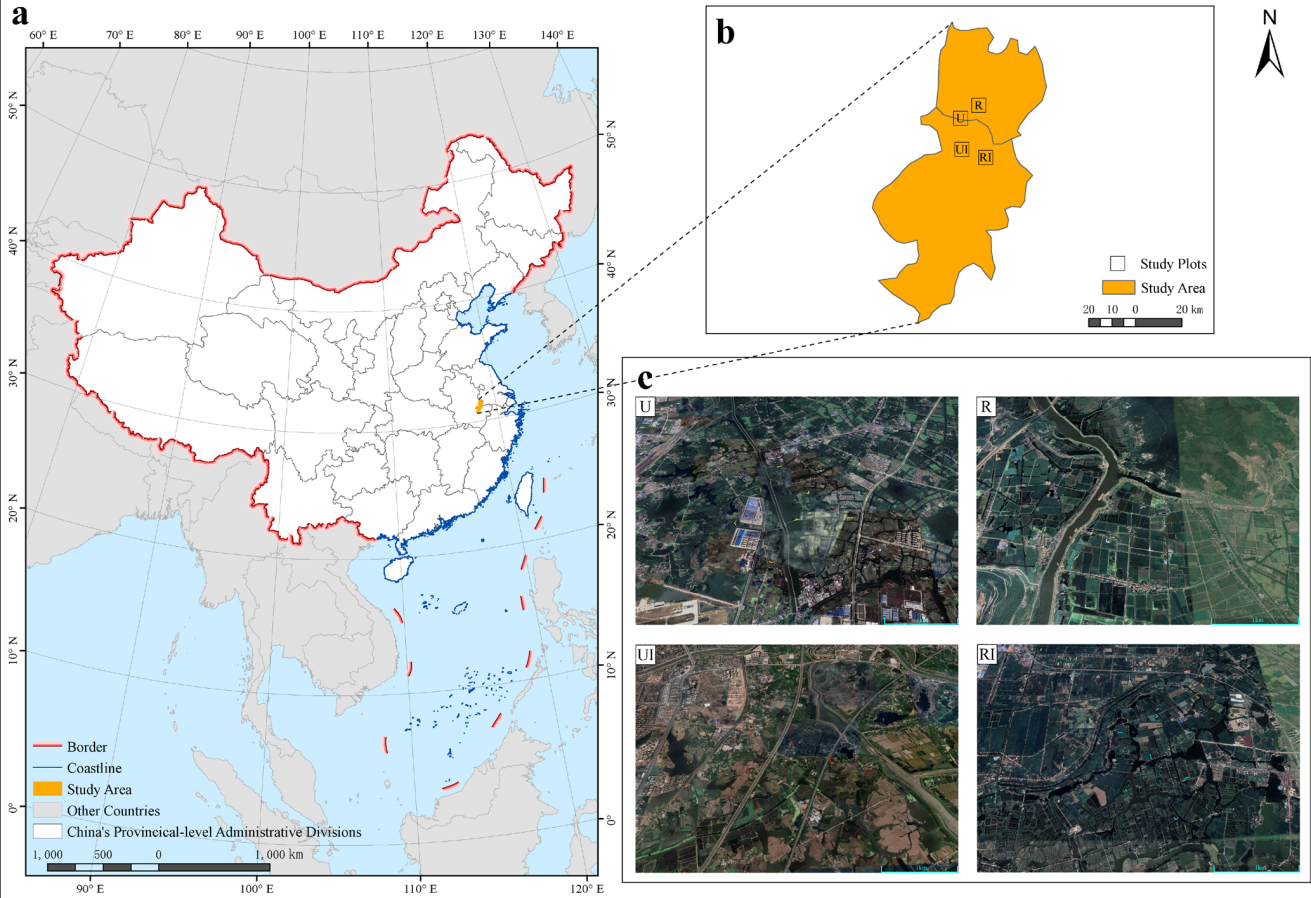
Study area schematic diagram. (a) Displays China schematic diagram. (b) Displays study area schematic diagram. (c) Displays four research areas local image maps: urban bullfrog invaded area (UI), urban bullfrog non‐invaded area (U), rural bullfrog invaded area (RI), and rural bullfrog non‐invaded area (R).

We established four comparative groups based on urbanization gradients (urban vs. rural) and bullfrog invasion status (invaded vs. uninvaded) (Figure 1): urban invaded (UI), urban uninvaded (U), rural invaded (RI), and rural uninvaded (R) sites. Invasion status was confirmed through preliminary surveys: invaded sites required documented breeding evidence (e.g., vocalizations or tadpole captures), while uninvaded sites showed no bullfrog detections for five consecutive years and were separated from invaded areas by natural barriers (e.g., ridges, highways). Amphibian surveys adopted the transect method, supplemented by vocal identification for bullfrogs (Melo et al. 2021; Heyer 1994). Seven fixed transects (100 × 2 m) were set up for each site type (28 in total) (Table S1), with a minimum distance of 500 m between adjacent transects. Surveys were conducted annually from 2020 to 2024 during the breeding season (May) and non‐breeding season (October), with three monthly replications per season. Each transect was surveyed by a team of two experienced observers. Observers moved along the center line at a prescribed pace of approximately 2 km/h to maintain consistent search intensity, while thoroughly searching all potential microhabitats (e.g., under vegetation, along the water's edge) and listening for vocalizations of the American bullfrog (MEE 2015; Wu et al. 2013). In addition to the positive identification, counting, and recording of all encountered anurans, a standardized set of environmental parameters was measured at the midpoint of each transect. The combined time required for the faunal census and environmental data collection amounted to approximately 25 min per transect. All surveys commenced 30 min after sunset to coincide with peak anuran activity (MEE 2015).

At the midpoint of each transect, environmental parameters were measured as follows: water depth (Hondex PS‐7 depth sounder); water temperature and dissolved oxygen (DO) (YSI ProODO); conductivity (Cond) and total dissolved solids (TDS) (Shanghai Leici DDBJ‐350). Vegetation cover (mean coverage) was quantified following GB/T 38590 using the diagonal intercept method, which recorded canopy closure (trees), coverage (shrubs and herbs), and composite community cover with a precision of 5% after accounting for overlapping strata (CNIS 2023).

### 2.2 Data Analysis

To investigate the spatiotemporal dynamics of bullfrog invasion and its ecological effects across urbanization gradients, we utilized R version 4.5.1 (R Core Team 2025) for all analyses. This integrated framework enabled robust examination of three core dimensions: (1) spatiotemporal invasion drivers, (2) native community impacts, and (3) anthropogenic modulation through pandemic restrictions. Primary analyzes employed generalized linear models (GLMs) with Tweedie distributions to accommodate the right‐skewed, continuous, and non‐negative characteristics of bullfrog density data (using the hist function to determine data distribution) (Thioulouse et al. 2018). We used the following key packages: glmmTMB (Brooks et al. 2017) for model fitting, DHARMa (Hartig 2024) for model diagnostics, car (Fox and Weisberg 2019) for variance inflation factors (VIFs), and MuMIn (Bartoń 2025) for multi‐model inference. For native species response assessment, we employed non‐parametric tests (Wilcoxon signed‐rank tests) and correlation analysis.

#### 2.2.1 Drivers of Invasion Patterns

This study aims to elucidate the spatiotemporal drivers of American bullfrog ( *Lithobates catesbeianus* ) invasion within urbanization contexts. Considering the nested data structure (repeated seasonal measurements along identical transects), we initially constructed a generalized linear mixed model (GLMM) as the foundational framework. This model specified bullfrog density as the response variable, with region type (Area: Urban vs. Rural) and season (Season: May vs. October) as fixed effects. A random intercept for transect lines (Lines) was incorporated to account for repeated measurement effects. To validate the model structure and assumptions, we performed comprehensive diagnostic checks. First, the significance of the random effect was assessed, revealing that its variance component approached zero (*σ* ^2^ < 0.001), indicating minimal explanatory contribution from between‐transect variations and justifying simplification to a GLM for parsimony. The final model was specified as: log(Density) ~ Area × Season with a Tweedie distribution (log‐link). We then rigorously tested the assumptions of this parsimonious model. Residual diagnostics were conducted using the DHARMa package, which confirmed compliance with normality (Kolmogorov–Smirnov test: *p* = 0.631) and homoscedasticity (dispersion test: *p* = 0.834). Additionally, variance inflation factors (VIFs) were calculated to assess multicollinearity among the fixed effects; all values were below 2, confirming its absence.

To evaluate potential influences of environmental factors, we first used the Kolmogorov–Smirnov test to determine the normality of all environmental factors (all *p* < 0.05), and then assessed collinearity among seven habitat variables (pH, water depth, vegetation cover, water temperature, total dissolved solids (TDS), conductivity, dissolved oxygen) using Spearman correlation analysis (Table 1). Results identified strong collinearity (|*r*| > 0.8) between water temperature and pH (*r* = −0.811), water depth (*r* = 0.836), and TDS (*r* = 1). Based on collinearity diagnostics, water temperature and TDS were excluded, retaining vegetation cover, pH, water depth, conductivity, and dissolved oxygen for subsequent analysis. These retained variables were standardized (*z*‐score) and evaluated for explanatory contributions through a model comparison framework: First, we established a base spatiotemporal model (Area × Season). Second, single‐environmental‐variable extension models were built (base model + individual environmental variables). Finally, a full‐environmental model was constructed (base model + all environmental variables). All models employed Tweedie distributions with log‐link functions. Model selection followed AICc criteria, supplemented by likelihood ratio tests to quantify additional explanatory value from environmental factors.

**TABLE 1:** Collinearity diagnostics of environmental predictors.

	pH	Depth	Vegetation cover	Temperature	TDS	Cond	Dissolved oxygen
pH	1.000	−0.738**	−0.554**	−0.811**	−0.233*	−0.234	0.652**
Depth	−0.738	1.000	0.648**	0.836	0.290**	0.292**	−0.667**
Vegetation cover	−0.554**	0.648**	1.000	0.562**	0.242	0.240*	−0.459
Temperature	−0.811**	0.836**	0.562	1.000	0.158	0.158	−0.734**
TDS	−0.233	0.290**	0.242*	0.158	1.000	1.000**	−0.053
Cond	−0.234	0.292**	0.240*	0.158	1.000**	1.000	−0.054
Dissolved oxygen	0.652**	−0.667	−0.459**	−0.734**	−0.053	−0.054	1.000

*Note:* “*” means *p* < 0.05; “**” means 0.001 < *p* < 0.01.

#### 2.2.2 Native Species Response Assessment

To evaluate the impacts of bullfrog invasion on native amphibian communities, we first quantified species‐specific responses using relative abundance ratios (density in invaded sites/density in uninvaded sites). These ratios were stratified by urbanization gradient (Urban vs. Rural) and breeding phenology (May vs. October) to capture spatiotemporal heterogeneity. Paired Wilcoxon signed‐rank tests were used to assess differences in responses between urban and rural areas for each species. Robustness was further verified through Spearman correlations between bullfrog density and native species densities, establishing a multidimensional assessment framework. Analyses focused on functionally divergent native species: the generalist *Bufo gargarizans* and four habitat‐specialized anurans— *Pelophylax nigromaculatus* , *P. plancyi* , *Fejervarya multistriata* , and *Microhyla fissipes* . *Rana zhenhaiensis* was excluded from analyses as it was absent in 98.2% of transects.

#### 2.2.3 Urban–Rural Differential Effects of Pandemic Phases

To quantify the differential modulation of American bullfrog invasion dynamics by the COVID‐19 pandemic phases and its interaction with the urban–rural gradient, we employed a GLM with a Tweedie distribution and log link function. The model structure was specified as: Density ~ Period × Area + Season. Here, Period (reference level: Lockdown, 2020–2022) captured the fundamental shift in human mobility regimes. Area (reference level: Rural) represented the critical urban–rural environmental gradient. The Period × Area interaction term directly tested our core hypothesis: that the pandemic‐induced reduction in human activity would disproportionately alter invasion patterns across urban and rural landscapes, potentially revealing underlying differences in their invasion potential. The categorical variable Season (May or October) was included as a fixed effect to account for breeding phenology. Prior to interpreting the model results, we conducted a series of diagnostic checks to validate its appropriateness. Using the DHARMa package, we simulated scaled residuals to assess the distributional assumptions. The results indicated that the Tweedie distribution effectively handled the properties of our density data, with diagnostic plots (QQ plot, residual vs. predicted) showing no major deviations from expectations. Formal tests confirmed no significant issues with dispersion (*p* = 0.976) or zero‐inflation (*p* = 1). Furthermore, variance inflation factors (VIFs) for all predictors were calculated and found to be below 5, confirming that multicollinearity was not a concern for parameter estimation.

Robustness check via multi‐model inference. To ensure that the estimated effect of the lockdown period was not confounded by short‐term meteorological conditions that may have co‐varied with the pandemic timeline, we extended our analysis to include key daily meteorological variables as covariates. Data on mean temperature (T2M), relative humidity (RH2M), precipitation (PRECTOTCORR), and solar radiation (ALLSKY_SFC_SW_DWN) were obtained from the NASA POWER database (https://power.larc.nasa.gov/) for each survey date. To determine the appropriate inclusion of these meteorological variables in subsequent analyses, we first assessed their distributional properties using the Kolmogorov–Smirnov test (ALLSKY_SFC_SW_DWN and PRECTOTCORR: *p* < 0.05; T2M and RH2M: *p* > 0.05). Given the presence of non‐normally distributed variables, Spearman correlation analysis was employed to evaluate collinearity. The analysis revealed no strong collinearity (All |*r*| < 0.8) among the four variables. Therefore, all four variables were retained, standardized (*z*‐score), and included in the multi‐model inference framework. We employed a multi‐model inference framework to objectively assess the potential contribution of these abiotic factors. This involved constructing a candidate set of 15 models encompassing all possible combinations of the four meteorological variables, thus systematically testing whether any combination of weather conditions could provide a better explanation for the observed patterns in bullfrog density than our core model. Model selection was based on the second‐order Akaike's Information Criterion (AICc), with models having ΔAICc < 2 considered to have substantial support. We further validated our inference using likelihood‐ratio tests comparing each extended model to the core model. For ecological interpretation of the model coefficients, we exponentiated the estimates: exp(β) quantified multiplicative effects on density (relative to the reference groups), while the interaction term coefficient exp(β_interaction) specifically quantified the differential effect of the pandemic phase (Post‐Pandemic vs. Pandemic) in urban areas relative to rural areas. The term 1—exp(β) was used to calculate the relative suppression rate where applicable.

## 3 Results

Systematic monitoring conducted in the Wuhu‐Ma'anshan border region from 2020 to 2024 revealed the spatiotemporal dynamics of American bullfrog ( *Lithobates catesbeianus* ) invasion and its differential impacts on native amphibian communities across urbanization gradients. Surveys covered four site types across 28 fixed transects, totaling 840 sampling events during the breeding (May) and non‐breeding (October) seasons. Alongside the invasive bullfrog, six native amphibian species were recorded: *Pelophylax nigromaculatus* , *P. plancyi* , *Fejervarya multistriata* , *Microhyla fissipes* , *Bufo gargarizans* , and *Rana zhenhaiensis* . *R. zhenhaiensis* was excluded from subsequent analyses due to near‐zero detection rates.

### 3.1 Spatiotemporal Patterns of Bullfrog Invasion and Urbanization Effects

**FIGURE 2:**
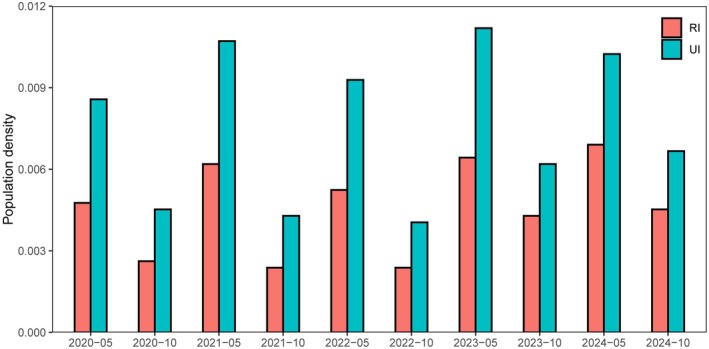
2020–2024 breeding season and non‐breeding season rural and urban America bullfrog invasion situation.

Systematic monitoring data revealed significant spatiotemporal heterogeneity in American bullfrog ( *Lithobates catesbeianus* ) invasion across urban–rural gradients. As shown in Figure 2, urban sites consistently exhibited higher bullfrog densities than rural sites across all survey periods (2020–2024) and seasons. Density fluctuations followed a seasonal pattern, with higher values observed during the breeding seasons (May) compared to non‐breeding periods (October). GLM analysis demonstrated that regional type and seasonal factors collectively shape bullfrog distribution patterns (Table 2). Bullfrog density in urban areas was significantly higher than in rural areas (estimate = 0.527 ± 0.110, *z* = 4.81, *p* < 0.001), with exponentiation indicating that urban density averaged 1.69 times rural density (exp(0.527) = 1.69). Seasonal fluctuations were equally pronounced, with non‐breeding season (October) density declining by 45% compared to breeding season (May) levels (estimate = −0.601 ± 0.139, *z* = −4.33, *p* < 0.001; exp(−0.601) = 0.55). The region‐by‐season interaction was non‐significant (estimate = −0.064 ± 0.179, *z* = −0.36, *p* = 0.72), indicating consistent urban–rural density differentials throughout the annual cycle.

**TABLE 2:** Estimates of fixed effects in the base model.

Variable	Estimate	SE	*z*‐value	*p*	AIC
Intercept	−5.132	0.085	−60.11	< 2e‐16***	
Area UI	0.527	0.11	4.81	1.52e‐06***	
Season October	−0.601	0.139	−4.33	1.50e‐05***	
Area UI: season October	−0.064	0.179	−0.36	0.72	
Model fit statistics					−777.6

*Note:* “***” means *p* < 0.001.

To evaluate potential environmental influences, we established a modeling framework incorporating vegetation cover, pH, water depth, conductivity, and dissolved oxygen. Model comparisons showed that while the vegetation cover–extended model achieved the lowest AICc value (−777.0), its improvement over the base model was marginal (ΔAICc = −0.5). The vegetation cover predictor exhibited a weak effect size (estimate = 0.091) and lacked statistical significance (*p* > 0.05), failing to substantially enhance model explanatory power. The likelihood ratio test comparing the full environment model to the base model showed no statistical significance (*χ* ^2^ = 5.272, df = 5, *p* = 0.384), indicating that adding environmental variables collectively did not significantly improve model fit.

Synthesizing model results, urban–rural differentials (urban > rural) and seasonal fluctuations (breeding > non‐breeding) constitute the core characteristics of bullfrog invasion patterns. This spatial and temporal structure operates independently of habitat variables including vegetation structure, water quality parameters (pH/conductivity/dissolved oxygen), and water depth.

### 3.2 Differential Impacts of Bullfrog Invasion on Native Species

**FIGURE 3:**
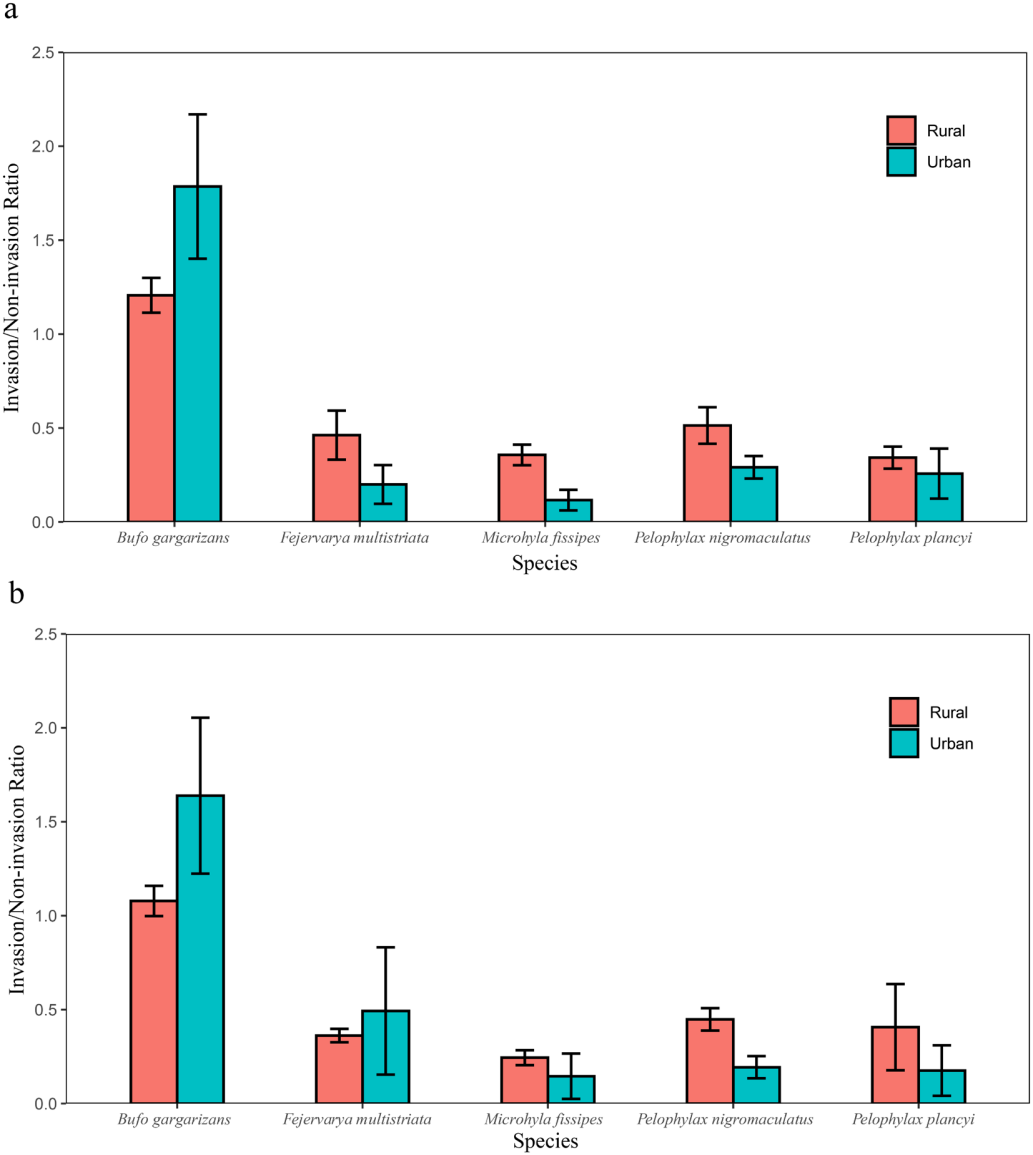
Breeding season (May) (a) and non‐breeding season (October) (b) 2020–2024 invasion effects on native species.

Invasion impacts exhibited strong species specificity (Figure 3). Relative abundance ratios (density in invaded sites/density in uninvaded sites), stratified by urbanization, revealed the following patterns: *Bufo gargarizans* displayed a unique positive response, with ratios consistently greater than 1 (breeding season: urban 1.786 ± 0.384, rural 1.207 ± 0.093; non‐breeding season: urban 1.639 ± 0.415, rural 1.079 ± 0.081). Urban ratios were significantly higher than rural ratios (*V* = 55, *p* < 0.01), suggesting that the presence of bullfrogs is positively associated with toad abundance, particularly in urban habitats. Conversely, all native frog species showed negative responses, with ratios predominantly less than 1. Importantly, except for *F. multistriata* , rural ratios were significantly higher than urban ratios for *P. nigromaculatus* , *P. plancyi* and *M. fissipes* (each *p* < 0.01), indicating intensified suppression of these species in urban landscapes. Correlation analyses further corroborated these findings (Table 3): bullfrog density was positively correlated with toad density but negatively correlated with the densities of all four frog species.

**TABLE 3:** Correlation analysis of invasion effects on native species population density.

Site season	Invasive species	BG	PN	PP	MF	FM
Urban‐5	LC	0.685**	−0.658**	−0.450*	−0.669**	−0.683**
Rural‐5	LC	0.645**	−0.523*	−0.567**	−0.571**	−0.621**
Urban‐10	LC	0.636**	−0.395	−0.439*	−0.122	−0.621**
Rural‐10	LC	0.581**	−0.485*	−0.376	−0.261	−0.672**

*Note:* “*” means *p* < 0.05; “**” means 0.001 < *p* < 0.01.

Abbreviations: BG, *Bufo gargarizans*; FM, *Fejervarya multistriata*; LC, *Lithobates catesbeianus*; MF, *Microhyla fissipes*; PN, *Pelophylax nigromaculatus*; PP, *Pelophylax plancyi*.

### 3.3 Modulatory Effects of COVID‐19 Lockdown and Reopening on Invasion Dynamics

The GLM quantifying the effects of the pandemic phase revealed a significant increase in bullfrog density following the reopening period compared to the lockdown (estimate = 0.354 ± 0.078, *z* = 4.52, *p* < 0.0001; Table 4). This core “period effect” underscores a strong response to the resurgence of human activity. The model also confirmed the persistent advantage of urban environments for bullfrogs, with densities significantly higher than in rural areas (estimate = 0.564 ± 0.064, *z* = 8.84, *p* < 0.0001). A pronounced seasonal fluctuation was evident, with density in October (non‐breeding season) being substantially lower than in May (breeding season) (estimate = −0.644 ± 0.048, *z* = −13.35, *p* < 0.0001). The interaction between Period and Area was not statistically significant (*z* = −1.37, *p* = 0.170). However, the negative coefficient for the interaction term (*β* = −0.129 ± 0.094) directionally suggested that the post‐reopening density increase was 12.1% weaker in urban areas compared to rural areas, implying a potential difference in the reliance on human‐mediated dispersal between landscapes.

**TABLE 4:** Before and after epidemic effects on invasion (America bullfrog population density) in different regions (urban; rural).

Variable	Estimate	SE	*z*‐value	*p*	AIC
Intercept	−5.273	0.053	−98.86	< 2e‐16***	
Period reopening	0.354	0.078	4.52	6.24e‐06***	
Area urban	0.564	0.064	8.84	< 2e‐16***	
Season October	−0.644	0.048	−13.35	< 2e‐16***	
Period reopening: area urban	−0.129	0.094	−1.37	0.17	
Model fit statistics					−643.6

*Note:* “***” means *p* < 0.001.

A multi‐model inference analysis was conducted to test the robustness of the central finding—the significant post‐reopening increase—against potential confounding by meteorological conditions. The results provided decisive support for the core model (Table S2). While the model incorporating mean air temperature (T2M) received the lowest AICc value (−642.0), the core model was statistically equivalent, falling well within the range of substantial empirical support (ΔAICc = 1.03, Akaike weight = 0.14). Critically, the core model was overwhelmingly superior to any model incorporating multiple meteorological variables. For instance, the model containing all four weather covariates (the full model) performed poorly (ΔAICc = 6.70, weight < 0.01). This pattern, where model performance decreased with increasing meteorological complexity, indicates that adding weather information led to overfitting rather than improved explanation. This conclusion was further validated by a comprehensive series of likelihood‐ratio tests, all of which confirmed that no model with meteorological covariates offered a significant improvement over the core model (all *p* > 0.05).

The analysis demonstrates that the significant “period effect” is a robust ecological signal. The systematic evaluation of meteorological variables confirms that the increase in bullfrog density following the relaxation of restrictions is most parsimoniously explained by the change in human activity levels itself, rather than by coincidental variation in weather conditions.

## 4 Discussion

Urbanization and biological invasions are dual engines of global amphibian decline. Our study leveraged the unique variation in human activity during the COVID‐19 pandemic to elucidate their interactive effects. We demonstrate that urbanization significantly facilitates American bullfrog invasion, amplifies its negative impacts on native frogs through mechanisms largely independent of local habitat quality, and modulates species‐specific responses. The post‐lockdown resurgence of human activity further provided robust evidence of its role in driving invasion dynamics. Below, we interpret these findings by focusing on the underlying mechanisms and their implications for conservation.

### 4.1 Urbanization as a Catalyst for Invasion

Consistent with the disturbance hypothesis (Jeschke and Heger 2018), bullfrog densities were 1.69 times higher in urban than rural sites—a pattern that could not be explained by measured habitat variables (e.g., vegetation cover, conductivity, temperature). This finding underscores that socioeconomic mechanisms, rather than local habitat quality, are the primary drives of bullfrog invasion success in urban areas. We posit three key pathways through which this occurs: first, anthropogenic dispersal networks (e.g., pet trade releases, religious practices, and transport hubs) provide efficient invasion corridors (Pyšek et al. 2020), with cities functioning as introduction hotspots; second, permanent aquatic habitats in urban areas (such as urban ponds and water features) support the life‐history needs of bullfrogs (Sawada and Kamijo 2024), while reduced predator pressure in cities further enhances their survival (Eötvös et al. 2018); and third, habitat fragmentation forces native amphibians into confined water bodies, which increases their encounter rates with bullfrogs and accelerates competitive exclusion.

These mechanisms align with global evidence identifying cities as invasion epicenters (Gaertner et al. 2017; McKinney 2008) but challenge paradigms that attribute amphibian declines to habitat degradation (Hamer and McDonnell 2008). Instead, human‐mediated dispersal and urban landscape structure emerge as dominant drivers. Furthermore, the urban landscape may exert novel evolutionary pressures, selecting for bullfrog individuals with traits like increased tolerance to pollution, bolder behavior in human presence, or altered reproductive timing, potentially leading to rapid local adaptation that further cements their advantage over native species (Alberti 2015).

### 4.2 Species‐Specific Impacts and the Role of Functional Traits

The stark contrast between the positive response of *Bufo gargarizans* and the declines observed in native frogs underscores that invasion outcomes are not uniform but are critically determined by the interplay between species' functional traits and the novel selective pressures of urban environments.

The success of *B. gargarizans* in invaded sites, particularly urban ones, likely stems from a suite of traits that reduce direct conflict with bullfrogs and enhance tolerance to human disturbance. As a habitat and dietary generalist, it may exhibit lower niche overlap with the invasive bullfrogs, minimizing competition. Furthermore, its documented resistance to pathogens such as *Batrachochytrium dendrobatidis* (Fu et al. 2023), potentially carried by bullfrogs, provides a key immunological advantage. This pattern aligns with the concept of exaptation, where pre‐existing traits are co‐oped for success in novel environments, allowing species that possess them to gain an advantage needed for coexistence where others fail (Poe et al. 2011). The behavioral flexibility of such generalists may also be crucial, as animals with high behavioral plasticity are more likely to adjust to novel situations, leading to a generalist‐opportunistic lifestyle (Klopfer 1967; Sol et al. 2013), allowing them to exploit resources in invaded and urbanized landscapes effectively.

Conversely, the decline of native frogs (*Pelophylax* spp., *Fejervarya multistriata* , *Microhyla fissipes* ) points to their higher vulnerability, likely driven by greater niche overlap with bullfrogs and a lack of compensatory traits. *Pelophylax* species, being ecologically similar congeners, may suffer intensely from larval competition (Kupferberg 1997), direct predation by adult bullfrogs (Kats and Ferrer 2003), and disease transmission (Wu et al. 2004). The intensified suppression of these species in urban habitats is a pivotal finding. This artificial aggregation of native amphibians, as driven by habitat fragmentation, creates a “disturbance‐competition‐disease” stressor triad that disproportionately filters out less tolerant species. This process effectively filters the community, favoring tolerant generalists and eliminating sensitive specialists, thereby accelerating biotic homogenization (McKinney 2006).

### 4.3 Human Activity Fluctuations and Invasion Dynamics

The COVID‐19 lockdown created a unique perturbation to assess the role of human mobility. A key concern is whether the “period” effect (Lockdown vs. Reopening) truly captures changes in human activity or is confounded by other factors, particularly seasonal weather. Our multi‐model inference analysis robustly addressed this by demonstrating that the significant post‐reopening increase in bullfrog density is best explained by the period itself, not by coincidental meteorological conditions. This core “period effect” underscores a strong response to the resurgence of human activity.

The differential response to the resumption of human activity reveals a fundamental disparity in the invasion process between urban and rural landscapes. We interpret this pattern through the ecological stages of urban colonization, which can be described as arrival, establishment, and increase/spread (Evans et al. 2010; Sol et al. 2013). This aligns with the unified framework for biological invasions, which posits that populations must overcome barriers to progress through these stages (Blackburn et al. 2011).

Our data suggest that urban bullfrog populations have transitioned to the latter establishment and increase stages. Their ability to maintain high densities even during the lockdown, when human‐mediated dispersal was restricted, indicates they have become self‐sustaining populations. This transition can be explained by the propagule pressure hypothesis (Lockwood et al. 2009), which posits that the probability of a population becoming self‐sustaining increases with the number of individuals introduced, as a larger founder pool is better able to buffer against demographic and environmental stochasticity and overcome Allee effects (Blackburn et al. 2009). The historically intense and chronic propagule pressure in urban areas, driven by factors like the pet trade and intentional releases, has thus likely provided the necessary numerical strength for urban bullfrog populations to cross this critical threshold (Lockwood et al. 2005).

In contrast, rural populations, characterized by lower baseline densities and a significantly stronger growth surge following the reopening, are indicative of populations in the earlier arrival and establishment stages. Their dynamics appear more tightly coupled with contemporary, pulse‐like human‐mediated dispersal events. The resumption of human activity likely reinstated key dispersal pathways (e.g., via vehicular transport or equipment movement) (Wilson et al. 2009), providing a fresh influx of propagules. This pattern underscores the role of propagule pressure as an immediate driver of population growth for nascent populations that have not yet achieved self‐sustaining status (Lockwood et al. 2005).

In ecological monitoring, the detectability of species is rarely perfect and may be affected by covariates. Therefore, if not taken into account, raw counts may produce biased estimates of true abundance or richness (KÉRy and Plattner 2007; Kéry and Schmid 2004). In our study, we must consider whether the higher bullfrog density observed after reopening reflects increased detectability rather than, or as well as, a real population increase. The post‐reopening increase in bullfrog density could theoretically be influenced by changes in detectability. For instance, human disturbance can alter animal behavior and thus potentially change species detectability. It might decrease it by making bullfrogs more alert (Frid and Dill 2002), or increase it if bullfrogs in frequently disturbed areas become habituated and thus less wary (Stankowich 2008). However, we argue that a true population increase is the most parsimonious explanation. First, our standardized protocol—nocturnal surveys by experienced observers along fixed transects—was explicitly designed to minimize variability in detectability arising from observer experience and diurnal activity patterns. Second, the secretive aquatic nature of bullfrogs makes a substantial, human‐induced increase in visibility through habituation unlikely over the short term. Most critically, a model relying solely on detectability shifts struggles to explain the spatially patterned response (i.e., the stronger trend in rural areas), whereas our proposed model of stage‐dependent invasion dynamics provides a coherent mechanism for the full pattern of results.

### 4.4 Conservation Implications

Our findings underscore urgent needs for targeted management strategies across different contexts and species: in urban areas, where tolerant invaders are likely already established, efforts should focus on containment and impact mitigation; disrupting bullfrog dispersal pathways through pet trade regulations, bans on religious releases, and restoration of habitat connectivity to reduce native species aggregation. In rural areas, which may serve as refugia for less tolerant species but are also vulnerable to new invasions, the priority should be prevention and early eradication. Vigilant monitoring and rapid response are essential to eliminate nascent bullfrog populations before they become self‐sustaining. Species‐specific approaches are paramount. Conservation resources should be prioritized for susceptible habitat specialists (e.g., *Pelophylax* spp.), while the ecology of tolerant species like *B. gargarizans* should be studied to understand potential mechanisms of coexistence.

## Author Contributions

**Jiayi Shi:** formal analysis (equal), investigation (equal), writing – original draft (equal). **Keming Wang:** investigation (equal). **Zhirong He:** investigation (equal). **Qingyan Sun:** investigation (equal). **Meiting Liu:** investigation (equal). **Suyue Wang:** investigation (equal). **Chunna Zhang:** investigation (equal). **Yujia Sun:** investigation (equal). **Na Zhao:** investigation (equal). **Supen Wang:** conceptualization (equal), formal analysis (equal), writing – original draft (equal).

## Ethics Statement

The animal study was reviewed and approved by the Anhui Normal University.

## Conflicts of Interest

The authors declare no conflicts of interest.

## Supporting information


**Table S1:** Line transect location information.

**Table S2:** Model selection table from the multi‐model inference analysis evaluating the contribution of meteorological variables. Models are ranked by AICc.

**Data S1:** ece372633‐sup‐0001‐DataS1.zip.

## Data Availability

The data that support the findings of this study are available from the Supporting Information: Data S1.
